# C-reactive protein promotes inflammation through TLR4/NF-κB/TGF-β pathway in HL-1 cells

**DOI:** 10.1042/BSR20190888

**Published:** 2019-08-28

**Authors:** Weiping Sun, Yongquan Wu, Mingyang Gao, Ying Tian, Peng Qi, Yujing Shen, Lihong Huang, Liang Shi, Yanjiang Wang, Xingpeng Liu

**Affiliations:** 1 Department of Cardiology, Beijing Chao-yang Hospital, Capital Medical University, Beijing 100037, China; 2 Department of Cardiology, Beijing Anzhen Hospital, Capital Medical University, Beijing 100029, China; 3 Department of Cardiology, Fuwai Hospital, Chinese academy of Medicine sciences, Beijing 100037, China; 4 Department of Cardiology, Beijing Luhe Hospital, Capital Medical University, Beijing 100037, China

**Keywords:** atrial fibrillation, inflammation, NF-κB, Toll-like receptor 4, transforming growth factor-beta1

## Abstract

Atrial fibrillation (AF) is the most common type of heart arrhythmia. Currently, the pathogenesis of AF is not fully understood yet. A growing body of evidence highlighted the strong association between inflammation and the pathogenesis of AF. C-reactive protein (CRP) is an inflammation marker with increased expression in AF. Therefore, the aim of this study was to determine if CRP promotes inflammation, which may sequentially mediate the onset of AF and the concurrent atrial fibrosis, through TLR4/NF-κB/TGF-β pathway. HL-1 cells were treated with either 25 or 50 μg/ml recombinant human CRP. TGF-β1 and NF-κB inhibitors were given either solely or together to the 50 μg/ml CRP-treated cells. Cell proliferation, apoptosis, the expression of apoptotic factors and TLR4, IL-6, TGF-β1, Smad2, and the phosphorylation of Smad2 were determined. Data showed that CRP induced dose-dependent inhibition on cell proliferation and promoted cell apoptosis, which was induced through both intrinsic and extrinsic pathways. Such effects were reversed by inhibiting TGF-β1 and/or NF-κB. Inhibition of TGF-β1 and/or NF-κB also reduced the expression of TLR4 and IL-6. Inhibition of NF-κB alone weakened the expression of TGF-β1 and phosphorylation of Smad2. Our study demonstrated that CRP is not only a marker, but also an important mediator in the induction of inflammation and likely the pathogenesis of AF. We for the first time reported CRP-induced activation and cross-talk between TLR4 and NF-κB/TGF-β1 signaling pathway in a cardiomyocyte model. Reducing CRP and targeting TLR4/NF-κB/TGF-β1 pathway may provide new insights in the therapeutic interventions to inflammation-induced AF.

## Introduction

Atrial fibrillation (AF) is the most common type of heart arrhythmia characterized by rapid and irregular heartbeat [1]. The Global Burden of Diseases, Injuries, and Risk Factors Study reported that from 2007 to 2017, there was a 21.1% increase in deaths caused by all types of cardiovascular diseases at all age groups globally, among which AF and atrial flutter had an increase of 47.8%—the second highest increase following peripheral vascular disease, which increased 55.7% in 10 years [2]. Currently, the pathogenesis of AF is not fully understood yet.

A growing body of evidence highlighted the strong association between inflammation and the pathogenesis of AF (reviewed by [3]). C-reactive protein (CRP) is an acute inflammatory protein that expression sharply elevates under inflammation and infection conditions [4]. Researchers have found that higher circulation CRP level is associated with increased risks of cardiovascular diseases, including cardiomyopathy [5], myocardial infarction [6], heart failure [7] and AF [3,8–10]. Meanwhile, immunotherapy and surgical interventions that reduce the recurrence of AF is accompanied with reduced circulation CRP, and increased CRP level post-intervention is a predictor of the recurrence of AF [11,12]. Moreover, recently, it is found that CRP can activate Toll-like receptor (TLR) 4/interferon regulatory factor-3/NF-κB pathway and induce the secretion of pro-inflammatory factor interleukin (IL)-6 to promote inflammation in rat vascular smooth muscle cells [13]. In turn, CRP binds to phosphatidylcholine-generating long-chain acylcarnitines and lysophosphatidylcholines under the presence of Ca^2+^ in AF, which is associated with the overload of Ca^2+^ and sequential cell membrane dysfunction, and eventually results in cell death (apoptosis) and may further exacerbate inflammation [8]. Thus, rather than a marker, CRP is also a mediator in the promotion and persistence of inflammation.

Persistent AF is associated with the volume and composition change of extracellular matrix, where accumulation of fibrillar collagen is observed to replace the degenerative tissue [14]. Increased deposition of collagen can sequentially lead to atrial fibrosis [14]. Atrial fibrosis can then induce abnormalities in conduction, which in turn, further exacerbate and sustain AF [15]. The transforming growth factor-β (TGF-β) signaling pathway is reported to play a central role in the regulation of atrial fibrogenesis [16]. TGF-β binds to the transmembrane TGF-β1 receptor to phosphorylate receptor-associated Smads [16]. Activation of this signaling pathway would therefore regulate the downstream transcriptions. Overexpression of TGF-β promotes atrial fibrosis and block of TGF-β reduces its occurrence [15].

In the glioblastoma cells, hyperactivation of NF-κB enhanced the strength and prolonged the activation of TGF-β/Smad signaling through the mediation of miRNAs and thus promotes the propagation of glioblastoma cells [17]. Though increased expression of TGF-β and NF-κB were observed in angiotensin II-induced inflammation [18] and during heart failure [19], it is not clear if CRP also induce increased expression of NF-κB and TGF-β to promote the onset of AF following a similar pattern as being observed in the glioblastoma cells. Since CRP is found to be positively correlated with the onset of AF, and CRP can activate NF-κB pathway through the mediation of TLR4 to induce the secretion of pro-inflammatory factors, NF-κB may also facilitate the enhanced and prolonged activation of TGF-β/Smad signaling in AF. If so, this CRP induced activation of TLR4/NF-κB/TGF-β pathway may provide new insights in the therapeutic interventions to inflammation-induced AF and concurrent atrial fibrosis. Therefore, the aim of the present study was to determine if CRP promotes inflammation, which may sequentially mediate the onset of AF and the concurrent atrial fibrosis, through TLR4/NF-κB/TGF-β pathway. We adopted the HL-1 cell line—the model cardiac cell with functional properties comparable to the normal cardiomyocytes, to determine (1) the effect of CRP on the proliferation and apoptosis of HL-1 cells; (2) the effect of CRP on the secretion of pro-inflammatory factor IL-6 hallmarked in AF; and (3) the role of NF-κB in TGF-β/Smad signaling upon CRP treatment.

## Materials and methods

### Cell line and treatment

The HL-1 cardiac muscle cell line (Sigma, MO, U.S.A.) was cultured in Claycomb medium supplemented with 10% fetal bovine serum, 1% penicillin/streptomycin, 0.1 mM norepinephrine and 2 mM L-glutamine at 37°C with 5% CO_2_. All supplements were obtained from Sigma. The recombinant human CRP protein was obtained from Invitrogen (CA, U.S.A.). The endotoxin level of CRP was determined by chromogenic Limulus amebocyte lysate assay (Lonza, MD, U.S.A.) and well controlled under 0.1 EU/ml. Cells were divided into six treatment groups: (1) control, (2) 25 μg/ml CRP, (3) 50 μg/ml CRP, (4) 50 μg/ml CRP + 5 μM Helenalin [NF-κB (p65) inhibitor; Santa Cruz, CA, U.S.A.], (5) 50 μg/ml CRP + 5 μM LY364947 (TGF-β1 inhibitor; R&D Systems Inc., MN, U.S.A.), (6) 50 μg/ml CRP + Helenalin + LY364947. Culture medium was changed daily. The concentration of NF-κB inhibitor was adopted from [20–22] and the concentration of TGF-β1 inhibitor was adopted from [23–26] with the preliminary tests indicating that these concentrations did not cause toxicity to the HL-1 cells.

### Cell proliferation assay

Cell proliferation was determined by Cell Counting Kit-8 (CCK-8; Dojindo, Tokyo, Japan). Prior to the experiment, cells were maintained in serum-free medium for 24 h to synchronize cell cycle, then seeded at a density of 2 × 10^3^ in 96-well plate in culture medium with above-mentioned treatments. Cell number was counted every 12 h post-treatment after 4-h incubation with 10% CCK-8 solution at 37°C, with the absorbance measured at 450 nm by plate reader. Cell proliferation was determined from three replicates in each treatment group.

### Cell apoptosis assay

Cell apoptosis was determined by terminal deoxynucleotidyl transferase-mediated dUTP nick-end labeling (TUNEL) assay using *In Situ* Cell Death Detection Kit, POD (Roche, Shanghai, China) following the manufacturer’s instructions. Cells were fixed by 4% paraformaldehyde in PBS (pH 7.4) and permeabilized with 0.1% Triton X-100 in 0.1% sodium citrate. Cell nuclei were counterstained by 4,6-diamidino-2-phenylindole (DAPI), which showed a blue color under the fluorescent microscope while the apoptotic cells were labeled by fluorescein isothiocyanate (FITC) and showed green fluorescence. Images were captured by Nikon Eclipse 80i fluorescent microscope. Views from 5 spots on each slide were captured and three replicates of each treatment were determined.

### Western blot

The cells were washed twice in PBS and then lysed with protein extracted by IP lysis buffer (Thermo Fisher Scientific). Protein concentration was determined by Pierce detergent compatible Bradford assay kit (Thermo Fisher Scientific). A total of 30 μg protein was loaded for separation by sodium dodecyl sulfate-polyacrylamide gel electrophoresis (SDS-PAGE) and were then transferred to a polyvinylidene difluoride membranes (Millipore, Billerica, U.S.A.). The membranes were blocked with 5% non-fat dry milk in Tris-buffered saline and then incubated with primary antibodies (anti-Caspase3, anti-Caspase8, anti-Caspase9, anti-cleaved Caspase3, anti-cleaved Caspase8, anti-cleaved Caspase9; Cell Signaling Technology, Shanghai, China and anti-TLR4, anti-IL-6, anti-TGF-β1, anti-pSmad2, anti-Smad2 and anti-GAPDH; Thermo Fisher Scientific) according to the manufacturer’s instructions, followed by incubation with horseradish peroxidase-conjugated secondary antibodies. The reactive bands were visualized by an enhanced chemiluminescence substrate solution (Millipore) according to the manufacturer’s instructions.

### Statistical analysis

Each experiment in the present study had three replicates. Data were collected from experiments in triplicate and analyzed with *t*-test or analysis of variance (ANOVA) by GraphPad Prism 7 (GraphPad, CA, U.S.A.). Data are presented as mean ± SD. *P* < 0.05 was considered significant.

## Results

### NF-κB and TGF-β inhibition reversed CRP-induced inhibition on HL-1 cell proliferation

**Figure 1:**
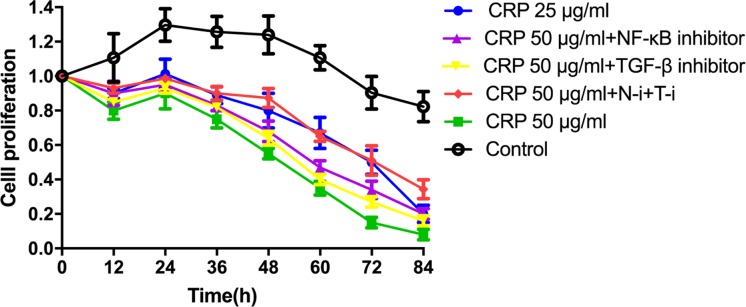
Effect of CRP on HL-1 cell proliferation The CCK-8 proliferation measurement initiated when cell reached 70% confluence (0 h). Three replicates were used in each treatment group. Data are shown as mean ± SD.

The effect of CRP on HL-1 proliferation is presented in Figure 1. The proliferation measurement started when cells grew to 70% confluence, which is corresponded with 1.0 on *Y*-axis in the figure. Cells reached 100% confluence at 24 h and then started to show contact inhibition. The HL-1 cells showed a dose-dependent response to CRP treatment. The 25 μg/ml CRP treatment decreased HL-1 cell proliferation from 36 h on post-treatment (*P* < 0.05), while the 50 μg/ml CRP treatment induced proliferation inhibition starting from 24 h post-treatment (*P* < 0.01). Adding either NF-κB or TGF-β1 inhibitor alone to 50 μg/ml CRP-treated cells did not reverse proliferation inhibition as compared to the 50 μg/ml CRP group. However, when both NF-κB and TGF-β inhibitors were added to the 50 μg/ml CRP-treated cells, proliferation inhibition was significantly reversed from 48 h on post-treatment compared to the 50 μg/ml CRP group (*P* < 0.05).

### NF-κB and TGF-β inhibition reversed CRP-induced HL-1 cell apoptosis

**Figure 2:**
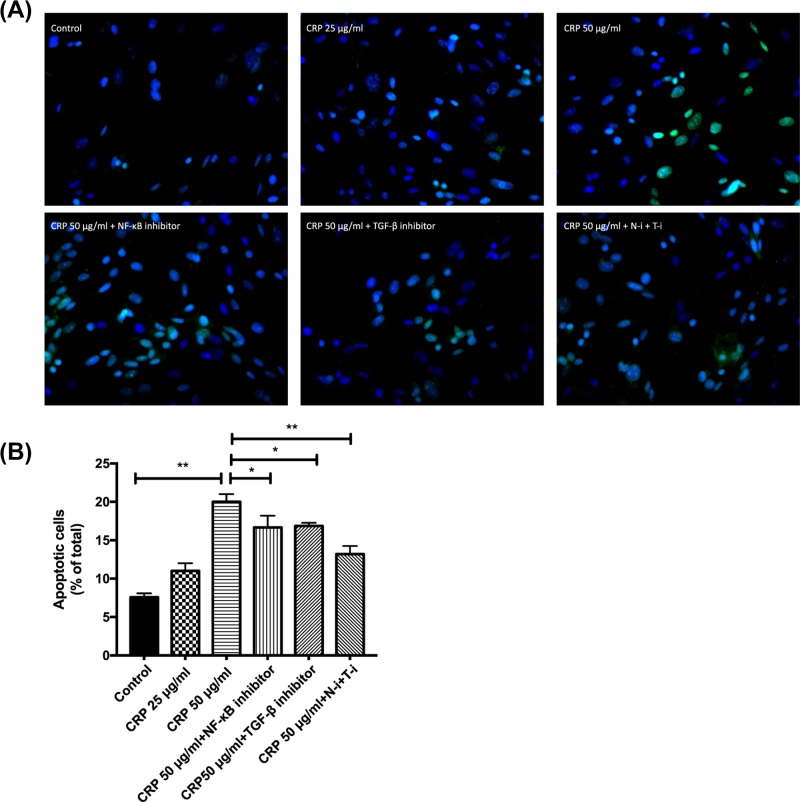
Effect of CRP on HL-1 cell apoptosis (**A**) Apoptotic cells (green fluorescence) under fluorescent microscope after TUNEL staining. (**B**) The apoptosis of cells treated with 25 or 50 μg/ml CRP were compared with the control cells, and apoptosis of cells treated with CRP + NF-κB and/or TGF-β inhibitors were compared with 50 μg/ml CRP-treated cells. Three replicates were used in each treatment group. Data are shown as mean ± SD; *P < 0.05, **P < 0.01.

**Figure 3:**
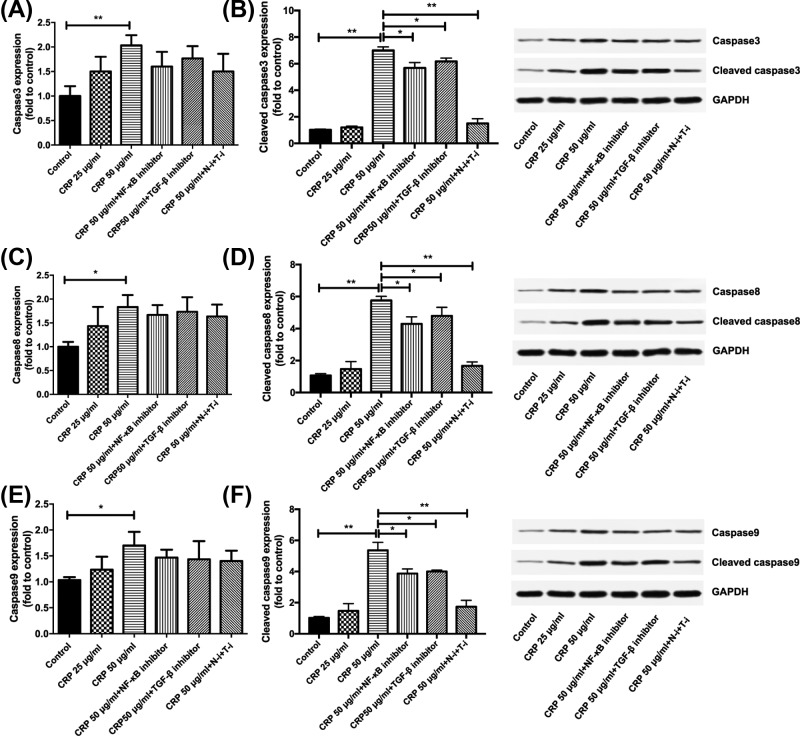
Relative expression of caspase enzymes upon CRP treatment (**A**) Caspase3, (**B**) Cleaved Caspase3, (**C**) Caspase8, (**D**) Cleaved Caspase8, (**E**) Caspase9 and (**F**) Cleaved Caspase9 expression in cells treated with 25 or 50 μg/ml CRP were compared with the control cells, and expression in cells treated with CRP + NF-κB and/or TGF-β inhibitors were compared with 50 μg/ml CRP-treated cells. Data are shown as mean ± SD; *P < 0.05, **P < 0.01.

The effect of CRP and NF-κB and TGF-β inhibition on cell apoptosis is shown in Figure 2A. In contrast with the effect on proliferation, the 25 μg/ml CRP treatment did not change cell apoptosis compared with the control group. The 50 μg/ml CRP treatment, however, significantly increased cell apoptosis (*P* < 0.01; Figure 2B). Though no effect was observed on proliferation when adding either NF-κB or TGF-β inhibitor alone, addition of either inhibitor to 50 μg/ml CRP-treated cells reduced apoptosis (*P* < 0.05). The highest reduction of 50 μg/ml CRP-induced apoptosis was achieved by adding both NF-κB and TGF-β inhibitors (*P* < 0.01). To further determine through which pathway was apoptosis induced by CRP, we measured the protein expression of non-cleaved and cleaved Caspase3, Caspase8 and Caspase9. The expression of all three enzymes, both non-cleaved and cleaved forms, increased upon 50 μg/ml CRP, but not 25 μg/ml CRP treatment (non-cleaved Caspase8 and Caspase9, *P* < 0.05; non-cleaved Caspase3 and cleaved enzymes, *P* < 0.01; Figure 3). Adding either NF-κB or TGF-β inhibitor to 50 μg/ml CRP-treated cells reduced the expression of cleaved Caspase3 (*P* < 0.01 and 0.05, respectively), Caspase8 (*P* < 0.01 and 0.05, respectively) and Caspase9 (*P* < 0.01), but not the non-cleaved enzymes. Adding both inhibitors to 50 μg/ml CRP-treated cells significantly reduced the expression of cleaved enzymes (*P* < 0.01), but not the non-cleaved enzymes.

### Inhibition of NF-κB and TGF-β alleviated CRP-induced inflammation

**Figure 4:**
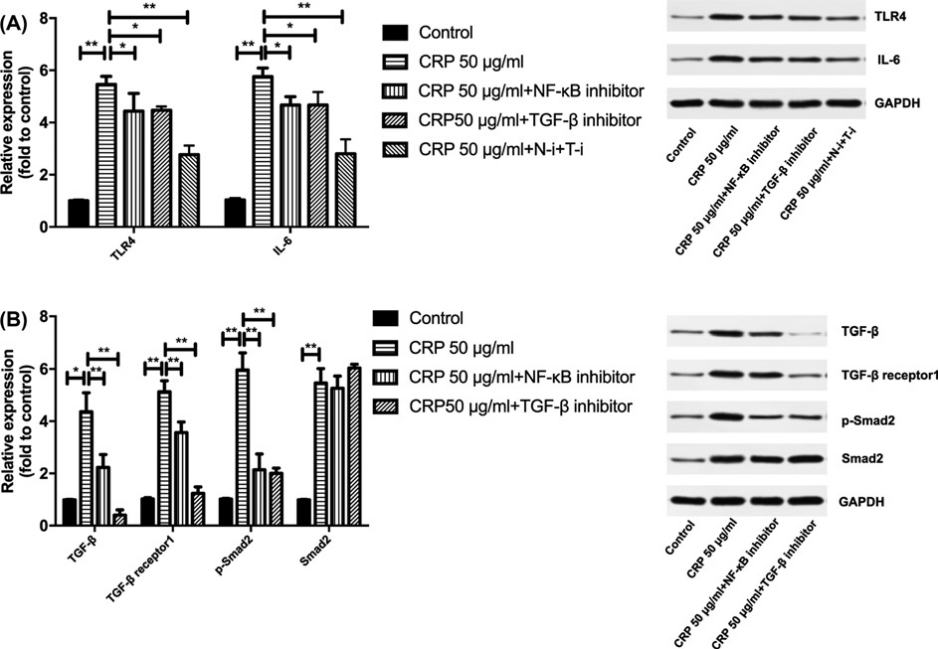
Effect of NF-κB and TGF-β inhibition on CRP-induced inflammation (A) and interaction between NF-κB and TGF-β (B) Glyceraldehyde-3-phosphate dehydrogenase (GAPDH) was used as the housekeeping protein. Data are shown as mean ± SD. Protein expression in cells treated with 50 μg/ml CRP was compared with the control cells, and expression in cells treated with CRP + NF-κB and/or TGF-β inhibitors were compared with 50 μg/ml CRP-treated cells; *P < 0.05,. **P < 0.01.

Treatment with 50 μg/ml CRP significantly increased the expression of TLR4 (*P* < 0.01) and IL-6 (*P* < 0.01; Figure 4A) as well as TGF-β (*P* < 0.01) and its receptor TGF-β1 (*P* < 0.01), Smad2 (*P* < 0.01) and phosphorylated Smad2 (*P* < 0.01; Figure 4B) in HL-1 cells. Inhibition of either NF-κB or TGF-β reduced the expression of TLR4 and IL-6 compared to that in 50 μg/ml CRP-treated cells (*P* < 0.05). Inhibition of both NF-κB and TGF-β reduced their expression the most (*P* < 0.01). To further determine if NF-κB plays a role in TGF-β signaling upon CRP treatment, we measured the expression of TGF-β and its downstream signaling factor Smad2 when NF-κB was inhibited under 50 μg/ml CRP administration. Data showed that the expression of TGF-β and its receptor TGF-β1, as well as the phosphorylation of Smad2 were lowered than the 50 μg/ml CRP group when NF-κB was blocked (*P* < 0.01).

## Discussion

CRP is one of the most commonly used markers for inflammation. Recently, an increasing body of evidence showed strong association between inflammation and the pathogenesis of AF [3,8,9,27]. Therefore, elevated CRP level is used as a prognostic marker for AF [9]. However, except for being a marker for AF, CRP per se is also found to trigger inflammation [13]. The aim of the present study was to determine if CRP promotes inflammation through TLR4/NF-κB/ TGF-β pathway, which may sequentially mediate the onset of AF and the concurrent atrial fibrosis. We used HL-1 cells, which were originally derived from mouse atrial cardiomyocyte tumor, as the atrial cardiomyocyte model. Our data showed that CRP had a dose-dependent effect on cell proliferation, and 50 μg/ml CRP treatment significantly reduced cell viability. We further explored through which pathway was cell apoptosis induced by CRP, and found that both Caspase8 and Caspase9 were upregulated during apoptosis. It should be noted that since CRP could both inhibit cell proliferation and promote apoptosis, it is not known its contribution to each proportion on the overall effect of the reduction of cell number. For instance, whether the unchanged cell number upon CRP treatment at 24 h was contributed by the compensation of apoptosis through increased cell proliferation is not clear. Protein expression data suggested that CRP indeed induced the increased secretion of pro-inflammatory factor IL-6 in HL-1 cells as well as the expression of TLR4. Moreover, CRP also upregulated the expression of TGF-β and its receptor TGF-β1. The downstream Smad signaling was thus intensified through increased phosphorylation. Inhibition of either NF-κB or TGF-β salvaged cell proliferation, reduced apoptosis, and alleviated TLR4-mediated inflammation, while the inhibition of both NF-κB and TGF-β showed the most significant interference to the effects of CRP on cell proliferation, apoptosis and inflammation. These results together, suggested that CRP per se could activate the NF-κB/TGF-β/Smad pathway to interfere cell viability, and induce inflammation through TLR4.

Previously, it is reported that uric acid-induced increase of CRP in human vascular smooth muscle cells (VSM) promotes cell proliferation and migration [28]. Further investigation revealed that recombinant CRP per se can already stimulate the proliferation of VSM to promote the onset of atherosclerosis [29], and this increase in proliferation is found to be mediated through the activation of NF-κB [13,30]. In addition, most recently, CRP is also found to promote the propagation of tongue squamous cell in the pathogenesis of tongue squamous cell carcinoma [31]. On the contrary, CRP inhibits proliferation, while induces apoptosis of human myeloid leukemia cell [31], which is in line with our observation in the HL-1 cells. These results together suggest that CRP can influence cell proliferation in the opposite directions dependent on the cell type. There is a scarce of data on the effect of CRP on either proliferation or apoptosis in the cardiomyocyte *in vivo* or *in vitro*. Our data here showed that CRP per se can induce apoptosis in HL-1 cells. CRP induces apoptosis in human coronary vascular smooth muscle cells through the activation of Caspase3 pathway [32], which supported our finding that CRP treatment lead to increased Caspase3 expression. There are three major pathways in cell apoptosis: (1) the intrinsic pathway, which is accompanied with mitochondria changes, is mediated through Caspase9; (2) the extrinsic pathway, which is dependent on the binding of death ligand and death receptor, is mediated through Caspase8; and (3) the granzyme pathway is mediated through perforin [33]. All three pathways, except for the granzyme A-mediated apoptosis, are executed by Caspase3 [33]. The fact that the expression of all three caspase enzymes were increased suggest that CRP-induced apoptosis in HL-1 cells was likely mediated through both intrinsic and extrinsic pathways.

Similar to what was found in the vascular smooth muscle cells [13], recombinant CRP also induced the expression of TLR4 and IL-6 in the HL-1 cells. TLR4 plays an essential role in the mediation of inflammatory responses in vascular diseases [34]. Increased TLR4 expression is found to be correlated with the new onset of AF [35]. TLR4 is found to induce the production of several pro-inflammatory factors, including IL-6, through the activation of NF-κB in both epithelial cells and macrophages [36,37]. And IL-6 in turn, can cross-talk with TLR4 to modulate the inflammatory responses [38]. CRP can also induce the expression of IL-6 in vascular smooth muscle cells and macrophages [13,39], and increased secretion of IL-6 can further induce the upregulation of CRP expression [40]. It is reported that CRP-induced secretion of IL-6 in the vascular smooth muscle cells is mediated through TLR4 [13]. As mentioned above, TLR4 stimulates the production of IL-6 by activating NF-κB. Meanwhile, TLR4 also promotes fibrosis through the augmentation of TGF-β activity [41]. Atrial fibrosis is one critical risk factor of AF and TGF-β1 is a well characterized mediator in the development of atrial fibrosis [14]. TGF-β stimulation precedes the nuclear translocation of NF-κB in colon cancer cells and sequentially activates NF-κB by binding to TGF-β1 receptor [42], while NF-κB enhances the strength and prolongs the duration of TGF-β/Smad signaling in glioblastoma [17]. In human endothelial cells, CRP is also found to activate NF-κB to induce atherogenic effects [43]. Therefore, we postulated that there is a cross-talk between NF-κB and TGF-β1 in CRP-induced inflammation in HL-1 cells. Results showed that inhibition of either NF-κB or TGF-β1 weakened the effect of CRP on proliferation inhibition and apoptosis induction, with the most intensified weakening by adding both inhibitors. Meanwhile, though both NF-κB and TGF-β1 are regulated by TLR4 upon inflammatory responses, inhibition of NF-κB and TGF-β1 also reduced the expression of TLR4 and IL-6, indicating that rather than a one-direction stimulation of NF-κB and TGF-β1 by TLR4, there is likely a cross-talk between NF-κB/TGF-β1 and TLR4 to regulate the inflammatory response upon CRP treatment. Furthermore, inhibition of NF-κB alone reduced the expression of TGF-β1 and the downstream phosphorylation of Smad2, suggesting that in cardiomyocytes, NF-κB may also play a role in regulating TGF-β1 signaling in CRP-induced inflammation.

Although the results from HL-1 cardiomyocyte model suggested that CRP promotes inflammation by activating TLR4/NF-κB/TGF-β1 pathway, it should be noted that the HL-1 cell line used in the present study was originally derived from mouse atrial cardiomyocyte tumor [44], which may be different from real human cardiomyocytes. Meanwhile, the atrial and ventricle myocytes have different metabolic physiology in cardiovascular diseases [45,46]. Therefore, whether the results in the present study are applicable to the ventricular myocytes requires further confirmation.

## Conclusions

In summary, our study demonstrated that CRP is not only a marker, but also an important mediator in the induction of inflammation, which may further mediate the pathogenesis of AF. CRP per se is sufficient to activate TLR4 and induce its sequential secretion of pro-inflammatory factors. TLR4 and NF-κB/TGF-β1 cross-talk to stabilize inflammatory signaling and promote cell death upon CRP treatment. The present study showed CRP-induced activation and cross-talk of TLR4/NF-κB/TGF-β1 signaling pathway in a cardiomyocyte model, which may provide a potential target for future therapeutic interventions in inflammation-induced atrial fibrillation.

## Highlights

- CRP decreased HL-1 cell proliferation and increased cell apoptosis in a dose-dependent manner.
- Inhibition of NF-κB and TGF-β reversed such effects of CRP and alleviated TLR4-mediated secretion of pro-inflammatory factor IL-6.
- There is a cross-talk between TLR4 and NF-κB/TGF-β1 signaling pathway upon CRP treatment.

## Abbreviations

AF: atrial fibrillation
CRP: C-reactive protein
FITC: fluorescein isothiocyanate
TLR: Toll-like receptor
