# Expression of Concern: C-reactive protein promotes inflammation through TLR4/NF-κB/TGF-β pathway in HL-1 cells

**DOI:** 10.1042/BSR-2019-0888_EOC

**Published:** 2024-04-24

**Keywords:** atrial fibrillation, inflammation, NF-κB, Toll-like receptor 4, transforming growth factor-beta1

The Editorial Office has been made aware of potential issues surrounding the scientific validity of this paper, hence has issued an expression of concern to notify readers whilst the Editorial Office investigates. It has been noted that Figure 2A CRP 25 µg/ml panel also seems to appear as Figure 7D miRNA-22-3p-inhibitor Merge panel in a paper by Zhou et al 2019 (DOI: 10.2147/ott.s224521).

